# Dynamics of Polymer Translocation: A Short Review with an Introduction of Weakly-Driven Regime

**DOI:** 10.3390/polym8120424

**Published:** 2016-12-07

**Authors:** Takahiro Sakaue

**Affiliations:** 1 Department of Physics, Kyushu University, Fukuoka 819-0395, Japan; sakaue@phys.kyushu-u.ac.jp; Tel.: +81-92-802-4066; 2 Precursory Research for Embryonic Science and Technology (PRESTO), Japan Science and Technology Agency, Honcho Kawaguchi, Saitama 332-0012, Japan

**Keywords:** polymer translocation, memory effect, generalized Langevin equation, nonequilibrium dynamic, tension propagation

## Abstract

As emphasized in a recent review (by V.V. Palyulin, T. Ala-Nissila, R. Metzler), theoretical understanding of the unbiased polymer translocation lags behind that of the (strongly) driven translocation. Here, we suggest the introduction of a *weakly-driven regime*, as described by the linear response theory to the unbiased regime, which is followed by the strongly-driven regime beyond the onset of nonlinear response. This provides a concise crossover scenario, bridging the unbiased to strongly-driven regimes.

## 1. Introduction

Macromolecules can be transported through a nanoscale pore by threading it. This process of *polymer translocation* has been extensively studied for the last two decades [1,2,3,4,5,6,7,8]. The motivation for the research arises from its relevance to biopolymer transport in living cells, and most notably, its connection to the nanopore-based new genome sequencing technique [9].

There are various important factors in the problem, including the electrokinetic effect, the pore properties (size, geometry, and interaction with polymer, etc.), and the capture process of polymer into the pore. Among others, one can ask how to characterize the very process of the polymer going through a simple pore, which is a purely a problem of polymer dynamics. The central quantity here is the translocation time *τ* as a function of the chain length *N* and the driving force *f*. Many efforts have been devoted to clarifying the scaling formula of *τ*, which is expected to be universal for long enough polymers [7,8].

**Figure 1:**
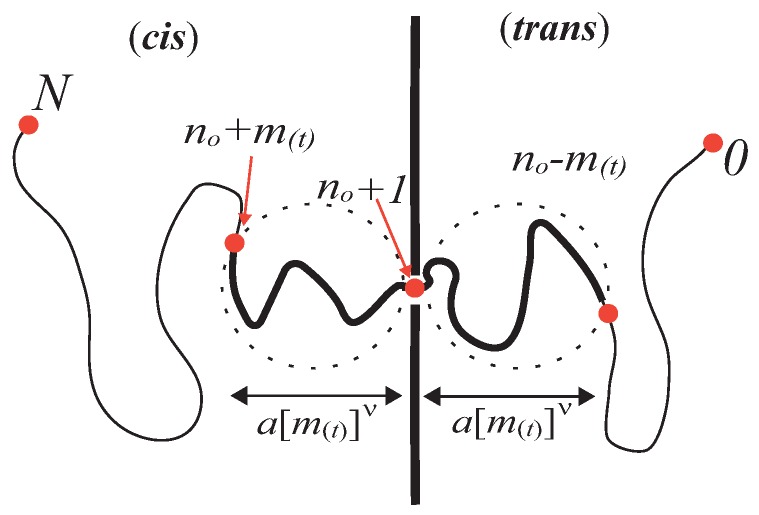
Illustration of a translocating polymer and the transport operation (Δn=1) to measure the stress relaxation associated with the tension propagation. The translocation coordinate is defined as the monomer label n(t) at the pore, which counts the number of monomers already in the *trans* side at time *t* in analogy to the reaction coordinate in chemical reaction process [7,8,23,24].

In the literature, the situation is categorized either by unbiased (f=0) or driven (finite *f*) translocation, depending on the absence or the presence of a driving force. In either case, an essential aspect in the problem lies in the collective dynamics of polymer associated with the tension propagation, which is manifested by the anomalous dynamics of the translocation coordinate (see Figure 1). In the present paper, we shall introduce the weakly-driven translocation regime in between. As will be shown, this provides a concise crossover scenario connecting three regimes. In Section 2, we first recall the memory effect approach to the unbiased translocation proposed by Panja, Barkema, and Ball [10]. In Section 3 , we then construct the weakly-driven regime through the linear response analysis to the unbiased regime. For larger driving force beyond the onset of nonlinear response $f>k_{B}T/\left(aN^{\nu }\right)$ (*a*: monomer size, $k_{B}T$: thermal energy), we enter the strongly-driven regime—the basic features of which are, by now, well understood. (For early theoretical and numerical attempts, see References [11,12,13,14,15]. The subsequent revision to the approach in References [11,12,13] can be found in References [16,17,18,19,20,21,22].) We shall briefly review it in Section 4. Summary and discussions are given in Section 5.

## 2. Unbiased Translocation

We first summarize self-similar dynamical properties of a flexible polymer, which will be used in subsequent discussions. Consider a partial section (with m<N bonds) of a long polymer. Its characteristic spatial size is $R_{m}≃am^{\nu }$, with ν≃3/5 in 3-dimensional space. Associated with it is the characteristic time $\tau_{m}≃\tau_{0}{\left(R_{m}/a\right)}^{z}$, where $\tau_{0}$ is a monomer time scale, and the dynamical exponent is $z=2+\nu^{-1}$ in the case of free-draining dynamics (see Appendix A.1).

Suggested from these two relations is

(1) $$\begin{array}{c} m\left(t\right)≃{\left(t/\tau_{0}\right)}^{1/(\nu z)} \end{array}$$

which describes how the tension created by perturbations propagates along the chain. By setting $m(\tau_{p})≃N$, we find the propagation time

(2) $$\begin{array}{c} \tau_{p}≃\tau_{0}N^{\nu z} \end{array}$$

at which the tension reaches the chain end. This is nothing but the longest time $\tau_{N}$ for the conformational relaxation.

### 2.1. Memory Function for Stress Relaxation

Panja et al. suggested that—due to the monomer exchange across the pore—there exists an imbalance in the tension near the pore, which is responsible for the subdiffusion of the translocation coordinate [10]. The problem can thus be naturally analyzed within the framework of the linear response theory. Consider a polymer going through a narrow pore from the head (n=0). Assume the polymer is in equilibrium with $n_{0}$ bonds in the *trans* side of the membrane, while the remaining $N-n_{0}$ bonds are on the *cis* side; i.e., the monomer’s label at the pore is $n\left(t\right)=n_{0}\;\left(t<0\right)$ (Figure 1). This may be realized by letting the polymer equilibrate with the immobilization constraint imposed on the monomer $n_{0}$ at the pore. Then, at t=0, we instantly transport Δn monomer from *cis* to *trans*. By this operation, the translocation coordinate n(t) is changed from $n_{0}$ to $n_{0}+\Delta n$. The polymer in the *trans* side is compressed, while it is stretched in the *cis* side, which produces the restoring force to the translocation coordinate.

To keep the imposed change in the translocation coordinate $n\left(t\right)=n_{0}+\Delta n$ at t>0, the force is required whose magnitude decreases with time along with the conformational relaxation. This process can be analyzed by the force balance equation

(3) $$\begin{array}{c} \int_{t_{0}}^{t}ds\;\Gamma \left(t-s\right)v\left(s\right)=f\left(t\right) \end{array}$$

where v(t)=dn(t)/dt, and we may set the lower bound of the time integral as $t_{0}\to -\infty$ by assuming the system is already in the equilibrium state before the operation is made. In the case of step displacement Δn imposed at t=0 (i.e., n(t+0)=n(t−0)+Δn), we have v(t)=Δnδ(t). The above equation is simplified to

(4) $$\begin{array}{c} \Delta n\Gamma (t)=f(t) \end{array}$$

To evaluate the memory kernel Γ(t), it is important to realize that the entire chain cannot respond to the operation at once. At time $t<\tau_{p}$, only a finite section with m(t) bonds given by Equation (1) close to the pore can respond to the operation. The deformation of such a responding chain section on the *trans* side is evaluated as $\Delta R_{m}=am{\left(t\right)}^{\nu }-a{\left\{m(t)+\Delta n\right\}}^{\nu }≃-a\left[m{\left(t\right)}^{\nu -1}\Delta n\right]\nu$ (Figure 1). The responding section on the *trans* side is thus compressed to the amount $∼a[m{\left(t\right)}^{\nu -1}\Delta n]$, which exerts a restoring force to the translocation coordinate. The magnitude of the force can be evaluated by noting that the responding domain with m(t) bonds acts as an entropic spring with the spring constant $≃k_{B}T/R_{m}{\left(t\right)}^{2}$. We thus find [10]

(5) $$\begin{array}{ccc} f(t) & ≃ & \frac{k_{B}T}{R_{m}{\left(t\right)}^{2}}\left|\Delta R_{m}\right|\\ & ≃ & \frac{k_{B}T}{am{\left(t\right)}^{1+\nu }}\left[\nu \Delta n\right]\\ & ≃ & \frac{k_{B}T}{a}\nu \Delta n{\left(\frac{t}{\tau_{0}}\right)}^{-(1+\nu )/(\nu z)} \end{array}$$

where Equation (1) is used in the last line. For the quantitative discussion, one should note that the same force arises due to the *cis* side stretching, so the net result doubles.

Comparison of Equations (5) with (4) yields the memory kernel

(6) $$\begin{array}{c} \Gamma \left(t\right)≃\frac{k_{B}T}{a}\nu {\left(\frac{t}{\tau_{0}}\right)}^{-\alpha } \end{array}$$

with the stress relaxation exponent

(7) $$\begin{array}{c} \alpha =\frac{1+\nu }{\nu z} \end{array}$$

In general, we have 0<α<1, reflecting the viscoelastic nature of the response.

### 2.2. Unbiased Translocation Dynamics

To connect the average stress relaxation with the subdiffusion of the translocation coordinate, we need to look at each realization of the stochastic processes. One is then led to the generalized Langevin equation by adding the thermal noise term ξ(t) to the right-hand side of Equation (3), which is zero mean 〈ξ(t)〉=0, and related to the memory kernel via the fluctuation-dissipation theorem $\langle \xi \left(t\right)\xi \left(s\right)\rangle =k_{B}T\Gamma (|t-s|)$. The equivalent expression of the generalized Langevin equation is

(8) $$\begin{array}{c} v\left(t\right)=\int_{t_{0}}^{t}ds\;\mu \left(t-s\right)f\left(s\right)+\eta \left(t\right) \end{array}$$

with the mobility kernel $\mu \left(t\right)∼-t^{\alpha -2}$ and the noise η(t), which again is related to the kernel as $\langle \eta \left(t\right)\eta \left(s\right)\rangle =k_{B}T\mu (|t-s|)$. In the unbiased case f(t)=0, the mean-square displacement (MSD) can be derived after integration of the velocity correlation function 〈v(t)v(s)〉=〈η(t)η(s)〉 twice with respect to time, yielding $\langle \delta n{\left(t\right)}^{2}\rangle ∼t^{\alpha }$, where δn(t)=n(t)−n(0); i.e., the stress relaxation exponent is equal to the MSD exponent. Therefore, the MSD in translocation coordinate space is obtained as

(9) $$\begin{array}{c} \langle \delta n{\left(t\right)}^{2}\rangle ≃{\left(t/\tau_{0}\right)}^{\left(1+\nu )/(\nu z\right)} \end{array}$$

### 2.3. Traveled Fraction at $t=\tau_{p}$

The memory kernels obtained for the translocating polymer arise from the viscoelastic response of the polymer due to the tension propagation. Therefore, the memory persists up to the propagation time $\tau_{p}$ given in Equation (2). To find how $\tau_{p}$ is related to the average time *τ* for the translocation process, let us calculate the characteristic displacement of the translocation coordinate at the time scale $\tau_{p}$. We find from Equation (9)

(10) $$\begin{array}{c} \langle \delta n{\left(\tau_{p}\right)}^{2}\rangle ≃N^{1+\nu }\ll N^{2} \end{array}$$

Now let us define the quantity

(11) $$\begin{array}{c} Q_{N}\equiv \frac{\sqrt{\langle \delta n{\left(\tau_{p}\right)}^{2}\rangle }}{N} \end{array}$$

which measures a fraction of the total chain length, which is traveled by the time $t=\tau_{p}$. Using Equation (10), we find

(12) $$\begin{array}{c} Q_{N}∼N^{-(1-\nu )/2}\ll 1 \end{array}$$

is vanishingly small for asymptotically large *N*. This indicates that the majority of the monomers do not pass the pore by $t=\tau_{p}$; i.e., $\tau \gg \tau_{p}$, so there should be another process to characterize the translocation dynamics. This requires us to think about the post-propagation stage at $t>\tau_{p}$.

### 2.4. Post-Propagation Stage

At $t>\tau_{p}$, the tension has already been propagated up to the chain end, so the motion of all the chain sections is coherent to generate the ordinary diffusion of the translocation coordinate. Here, the relevant question is what is the diffusion coefficient $D_{n}$ for it?

The following matching argument at $t=\tau_{p}$

(13) $$\begin{array}{c} \langle \delta n{\left(\tau_{p}\right)}^{2}\rangle ≃D_{n}\tau_{p}, \end{array}$$

where the left-hand side is evaluated by Equation (9), suggests

(14) $$\begin{array}{c} D_{n}≃\tau_{0}^{-1}N^{1+\nu -\nu z} \end{array}$$

The characteristic time $\tau_{pp}$ for the post-propagation stage is thus obtained as

(15) $$\begin{array}{c} \tau_{pp}≃{\left[N\left(1-Q_{N}\right)\right]}^{2}/D_{n}≃N^{1+(z-1)\nu } \end{array}$$

where the last near-equality is based on the estimation $Q_{N}\ll 1$. Since $\tau_{pp}/\tau_{p}\gg 1$, the scaling formula for the translocation time in the long chain limit becomes $\tau =\tau_{p}+\tau_{pp}∼\tau_{pp}$ [10].

## 3. Weakly-Driven Dynamics

The process may be biased by the external force. For translocating polymers, the force is acting at the pore, which is realized, for instance, by the voltage drop across the pore. As long as the force is weak enough $f<k_{B}T/R_{N}$, the tension dynamics summarized in the beginning of Section 2 is intact, and the generic linear response argument yields the average dynamics of the translocation coordinate. Suppose the system is already in equilibrium, and we start to apply a constant external force *f* at t=0. Equation (8) then simplifies to

(16) $$\begin{array}{c} \frac{d\langle n(t)\rangle }{dt}=f\int_{0}^{t}ds\;\mu \left(s\right)∼ft^{\alpha -1} \end{array}$$

where, as written after Equation (8), $\mu \left(t\right)∼-t^{\alpha -2}$, with 0<α<1. The average drift is thus evaluated as

(17) $$\begin{array}{c} \langle \delta n\left(t\right)\rangle ∼ft^{\alpha } \end{array}$$

### 3.1. Weakly-Driven Translocation Dynamics

From Equations (7) and (17), the average drift is

(18) $$\begin{array}{c} \langle \delta n\left(t\right)\rangle ∼ft^{\left(1+\nu )/(\nu z\right)} \end{array}$$

Therefore, the drift distance at the propagation time

(19) $$\begin{array}{c} \langle \delta n\left(\tau_{p}\right)\rangle ∼N^{1+\nu }f \end{array}$$

We thus find the fraction of the system explored by the propagation time

(20) $$\begin{array}{c} Q_{N}^{\left(f\right)}\equiv \frac{\langle \delta n\left(\tau_{p}\right)\rangle }{N}∼N^{\nu }f<1 \end{array}$$

where the last inequality comes from $f<k_{B}T/R_{N}∼N^{-\nu }$.

### 3.2. Post-Propagation Stage

The result $Q_{N}^{\left(f\right)}<1$ for a weakly-driven translocating polymer indicates that the translocation coordinate can explore only a fraction of the system by the propagation time. This conclusion is intact even with the fluctuation effect superimposed, since n(t)−〈n(t)〉 is (according to Equation (8)) described by the same dynamical equation as the unbiased dynamics, for which $Q_{N}<1$ as well. Therefore, the post-propagation stage at $t>\tau_{p}$ may become an essential part to determine the whole translocation time *τ*. Since the tension has already reached the chain ends at $t>\tau_{p}$, we expect the normal drift

(21) $$\begin{array}{c} \langle \delta n\left(t\right)\rangle ≃\frac{f}{\gamma_{n}}t\;\left(t>\tau_{p}\right) \end{array}$$

where the friction coefficient

(22) $$\begin{array}{c} \gamma_{n}∼N^{z\nu -1-\nu } \end{array}$$

can be determined by matching Equations (19) and (21) at $t=\tau_{p}$ (note the consistency of Equations (22) with (14) through Einstein relation). The characteristic time $\tau_{pp}$ for the post-propagation stage is thus found from the relation $\left(f/\gamma_{n}\right)\tau_{pp}=N-\langle \delta n\left(\tau_{p}\right)\rangle$; therefore,

(23) $$\begin{array}{c} \tau_{pp}≃\frac{\gamma_{n}}{f}N\left(1-Q_{N}^{\left(f\right)}\right)∼\frac{N^{(z-1)\nu }}{f} \end{array}$$

One can check the dominance of the post-propagation stage from the ratio $\tau_{pp}/\tau_{p}>1$ in the linear response regime. Comparing the two time scales $\tau_{pp}$ in unbiased and weakly-driven regimes, respectively, given by Equations (15) and (23), we find a characteristic force $f∼N^{-1}$, above which there exists a scaling regime of weakly-driven translocation where $\tau ≃\tau_{pp}$ given by Equation (23) applies. For weaker force f<1/N, the fluctuation effect dominates over the average drift, so we expect a crossover to unbiased scaling, where $\tau ≃\tau_{pp}$ given by Equation (15) holds.

## 4. Strongly-Driven Dynamics

As pointed out in Reference [11,12] and verified in subsequent works [14,15,17], the nonequilibrium dynamical effect becomes relevant for translocating polymer, which is driven by strong force. Suppose we grab one end of a polymer (with *N* monomers), and start to pull it by a constant force *f*. When the force is weak enough $f<k_{B}T/R_{N}$, the whole polymer will follow the force without noticeable conformational distortion. For a larger force, however, the polymer will be elongated along the direction of the pulling [25]. The first question here is the relation among the force, the moving velocity, and the elongation in steady state. Such a relation—which we may call a dynamical equation of state—is discussed in detail in Reference [26]. The second question is how we can characterize the transient process toward such an elongated steady state from an initial quiescent state. As one can infer from the scaling form of the threshold force $∼k_{B}T/R_{N}∼N^{-\nu }$, such a nonequilibrium effect shows up with rather moderate force; the “strongly-driven” appellation is only adopted to contrast to the weakly-driven regime discussed in Section 3.

Given a long relaxation time of the whole polymer $\tau_{N}≃\tau_{0}{\left(R_{n}/a\right)}^{z}≃\tau_{0}N^{\nu z}$, it would be intuitively clear that the polymer as a whole cannot respond to the pulling force all at once. Instead, only the subchain part close to the pulled site can initially respond, and thus collectively move in the pulled direction. Such a responding moving domain will grow with time, the dynamics of which is associated with how the tensile force propagates along the chain backbone [11,12,26] (see also [27]). To describe this sort of nonequilibrium response, it is useful to picture the whole polymer as composed of two distinct domains; a moving and a quiescent domain—the latter of which is yet unaware of the pulling force at a given moment. In this *two-phase picture*, we are interested in the dynamics of the tension front (i.e., domain boundary), which dictates the essential physics in the driven translocation.

**Figure 2:**
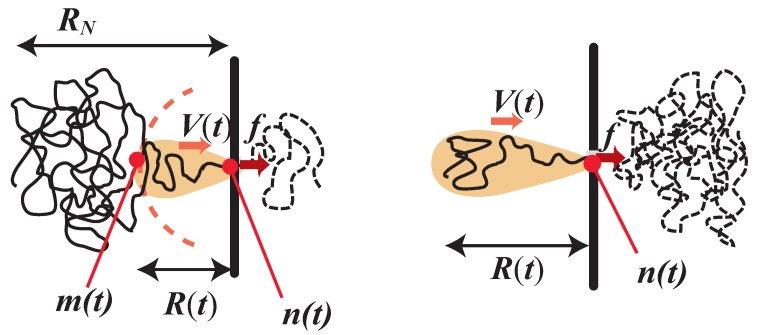
Sketch of a translocating polymer driven by a strong force *f*. (**left**) Propagation stage: a growing moving domain (with velocity V(t) and the size R(t)) on the *cis* side is shaded, while the chain portion already on the *trans* side is represented by a dashed curve. (**right**) Post-propagation stage: the tension has already reached to far end of the polymer; thus, m(t)=N is constant, and the moving domain is shrinking with time. In addition, most of monomers are already on the *trans* side, so this post-propagation stage adds a finite-size correction to the scaling formula of the translocation time.

Here we follow the argument in Reference [20] (see Appendix A.2). Now, in contrast to the weakly-driven regime (Figure 1), There appears significant dynamical asymmetry between *cis* and *trans* sides [28]. To set the stage, let us look at Figure 2. There is a thin wall at x=0 with a small pore, where the driving force with the constant magnitude $f>k_{B}T/R_{N}$ is locally exerted in the *x*-direction from the *cis* to the *trans* side. One chain end is initially sucked at time t=0. The translocation process, then, proceeds with the tension propagation along the chain. Monomers are numbered from the first sucked end to the other end (*N*th monomer). The moving domain at time *t* is specified by the monomer m(t) at the end of the moving domain, the monomer n(t) at the pore, the size R(t), and the representative (or average) velocity V(t). These can be determined by the following set of equations:

(24) $$\begin{array}{ccc} V(t)R(t) & ≃ & f^{z-2} \end{array}$$

(25) $$\begin{array}{ccc} m(t)-n(t) & ≃ & Rf^{-(1-\nu )/\nu }\;\left(≃R\sigma_{0}\right) \end{array}$$

(26) $$\begin{array}{ccc} m{\left(t\right)}^{\nu } & ≃ & R(t) \end{array}$$

As explained in Reference [13,26], Equations (24) and (25) are the dynamical equations of state describing the steady-state relation among velocity–extension–force (Equation (24)) and mass–extension–force (Equation (25)) for a dragged polymer. The information on the initial coiled conformation prior to the translocation process is contained in Equation (26). Note that $\sigma_{0}≃g_{f}/\xi_{f}≃f^{-(1-\nu )/\nu }$, where $\xi_{f}≃ag_{f}^{\nu }≃k_{B}T/f$ is the size of tensed blob in the close proximity of the pore, is the monomer line density at the pore, so that the monomer flux at the pore is

(27) $$\begin{array}{c} \frac{dn(t)}{dt}=j_{0}\left(t\right)=\sigma_{0}v_{0}\left(t\right) \end{array}$$

where $v_{0}\left(t\right)$ is the velocity of the monomer at the pore site.

To solve the above set of equations, we adopt the ansatz $v_{0}\left(t\right)=V\left(t\right)$ as in Reference [16,18], which amounts to correspond to the *iso-flux* condition [16,20]. Then, combining Equations (25) and (27), we obtain

(28) $$\sigma_{0}\left(V\left(t\right)+\frac{dR(t)}{dt}\right)≃\frac{dm(t)}{dt}$$

(29) $$\begin{array}{ccc} \Leftrightarrow & & f^{\left(z-2)-[(1-\nu )/\nu \right]}R{\left(t\right)}^{-1}≃R{\left(t\right)}^{(1-\nu )/\nu }\frac{dR(t)}{dt}-f^{-(1-\nu )/\nu }\frac{dR(t)}{dt} \end{array}$$

where Equations (24) and (26), and the expression for the line density at the pore (see Equation (25)) are used. This leads to the tension propagation law

(30) $$\begin{array}{c} R\left(t\right)∼{\left[tf^{\left(z-2)-[(1-\nu )/\nu \right]}\right]}^{\nu /(1+\nu )} \end{array}$$

which would be valid asymptotically under the condition $fR\left(t\right)/k_{B}T\gg 1$. The scaling formula for propagation time is thus obtained by setting $R\left(\tau_{p}^{\left(f\right)}\right)=R_{N}$;

(31) $$\begin{array}{c} \tau_{p}^{\left(f\right)}∼N_{0}^{1+\nu }f^{-(z-2)+[(1-\nu )/\nu ]} \end{array}$$

Note that the above propagation time can be written as

(32) $$\begin{array}{c} \tau_{p}^{\left(f\right)}∼\tau_{N}{\left(\frac{fR_{N}}{k_{B}T}\right)}^{1+(1/\nu )-z}<\tau_{N} \end{array}$$

where the last inequality—valid in the present condition $f>k_{B}T/R_{N}$—represents the fact that the tension propagation takes place in a time scale shorter than the conformational relaxation time, a clear indication of the nonequilibrium nature of the strongly-driven translocation process. Compare this result with the propagation time Equation (2) for equilibrium (unbiased and weakly-driven) regime.

After $t=\tau_{p}^{\left(f\right)}$, the post-propagation stage follows, which can be analyzed by Equation (28), but with dm(t)/dt=0 on the right-hand side [13]. A qualitative difference in the dynamical feature between propagation and post-propagation stages can be found in the growth rate of n(t); while the slowing-down in the propagation stage is a consequence of the growth of the moving domain, the speed-up in the post-propagation stage is due to the decrease of the overall friction with the process advanced. In contrast to the weakly-driven regime (Section 3), it can be shown that most of the monomers (in a scaling sense) are transported to the *trans* side in the propagation stage. Therefore, the scaling formula for the translocation time is given by the propagation time (i.e., $\tau \;≃\;\tau_{p}^{\left(f\right)}$) [11,12,16,19] (Note, however, that this does not hold for a semiflexible filament [29]).

To elucidate the crossover between weak and strongly-driven regimes, let us introduce the characteristic size of a tensed blob $\xi_{f}≃k_{B}T/f$ and the corresponding time scale $\tau_{f0}≃\tau_{0}{\left(\xi_{f}/a\right)}^{z}$. In the length scale smaller than $\xi_{f}$, the force is considered to be a weak perturbation. Therefore, under the force with the strength $k_{B}T/R_{N}<f<k_{B}T/a$, the physics in the weakly-driven regime applies up to $t=\tau_{f0}$, where the tension propagates according to Equation (1). The nonequilibrium effect and the *cis*–*trans* dynamical asymmetry manifests in larger length and time scales. In this sense, the length $\xi_{f}$ and time $\tau_{f0}$ play a role of “initial conditions” for the subsequent nonequilibrium dynamics. In this way, one can show that at $f>k_{B}T/R_{N}$, the tension propagation stage dominates the whole translocation process asymptotically; hence, a natural crossover occurs between two regimes at that force scale.

## 5. Summary and Discussions

### 5.1. Summary of the Scaling Formulae

As stated in Introduction, one of the main purposes of the present note is, aside from reviewing recent progress in the field, to introduce a weakly-driven regime as the linear response domain to the unbiased regime. To show that this provides a concise crossover scenario bridging the unbiased to the strongly-driven regime, let us summarize the scaling formulae for the translocation dynamics in different regimes, according to the classification scheme proposed here.

#### 5.1.1. Unbiased and Weakly-Driven Regimes

These two regimes may be termed collectively as the equilibrium regime. The statistical dynamics of the translocation coordinate—characterized here by its first and second moments of the displacement—follows

(33) $$\begin{array}{c} \langle \delta n\left(t\right)\rangle ∼\left\{\begin{array}{cc} ft^{\left(1+\nu )/(\nu z\right)} & \left(t<\tau_{p}\right)\\ \gamma_{n}^{-1}ft & \left(\tau_{p}<t<\tau \right) \end{array}\right. \end{array}$$

(34) $$\begin{array}{c} \langle {\left\{\delta n\left(t\right)-\langle \delta n\left(t\right)\rangle \right\}}^{2}\rangle ∼\left\{\begin{array}{cc} t^{\left(1+\nu )/(\nu z\right)} & \left(t<\tau_{p}\right)\\ D_{n}t & \left(\tau_{p}<t<\tau \right) \end{array}\right. \end{array}$$

where $\gamma_{n}∼N^{\nu z-1-\nu }$ and $D_{n}∼N^{1+\nu -\nu z}$ are the effective friction and diffusion coefficients of the translocation coordinate in the post-propagation stage. For unbiased case (f=0), the drift vanishes, so Equation (34) reduces to the MSD.

The translocation time is dominated by the post-propagation stage $\tau ∼\tau_{pp}$, with

(35) $$\begin{array}{c} \tau_{pp}∼\left\{\begin{array}{cc} N^{1+(z-1)\nu } & \left(f<N^{-1}\right)\\ N^{(z-1)\nu }/f & \left(N^{-1}<f<N^{-\nu }\right) \end{array}\right. \end{array}$$

Comparing the respective characteristic times of post-propagation stages, the characteristic force $f≃k_{B}T/\left(Na\right)$ is found; for weaker force, the bias is so weak that the process is essentially unbiased. Note that the same formula for *τ* was proposed in Equation (4) of Reference [12], based on the “equilibrium shape assumption”.

#### 5.1.2. Strongly-Driven Regime

As described in Section 4, for stronger force $f>k_{B}T/R_{N}$, one has to take the nonequilibrium conformational deformation dynamics into account [11,12]. In such a situation, the response becomes generally nonlinear, and the dynamical asymmetry between *cis* and *trans* sides appears [28]. This qualitatively changes the dynamics of tension propagation, hence the structure of the memory effect for the evolution of the translocation coordinate [11,12,13,14,15,16,17,18,19,20,21,22]. After the initial tensed blob of size $\xi_{f}≃k_{B}T/f$ in the vicinity of pore is formed (i.e., $t>\tau_{f0}$), its average time evolution is predicted to follow (see Appendix A.3)

(36) $$\begin{array}{c} \langle n\left(t\right)\rangle ∼\left\{\begin{array}{cc} t^{1/(1+\nu )}f^{\left(z-1-\nu^{-1}\right)/\left(1+\nu \right)} & \left(N^{-\nu }<f<N^{0}\right)\\ t^{1/(1+\nu )}f^{1/(1+\nu )} & \left(N^{0}<f\right) \end{array}\right. \end{array}$$

where the characteristic force $f≃k_{B}T/a$ separates the so-called trumpet and stem-flower regimes [25]. This process persists up to the propagation time $\tau_{p}^{\left(f\right)}$ given by $\langle m\left(\tau_{p}^{\left(f\right)}\right)\rangle ∼\langle n\left(\tau_{p}^{\left(f\right)}\right)\rangle ∼N$, which now depends on *f*;

(37) $$\begin{array}{c} \tau_{p}^{\left(f\right)}∼\left\{\begin{array}{cc} N^{1+\nu }f^{1+(1/\nu )-z} & \left(N^{-\nu }<f<N^{0}\right)\\ N^{1+\nu }f^{-1} & \left(N^{0}<f\right) \end{array}\right. \end{array}$$

**Figure 3:**
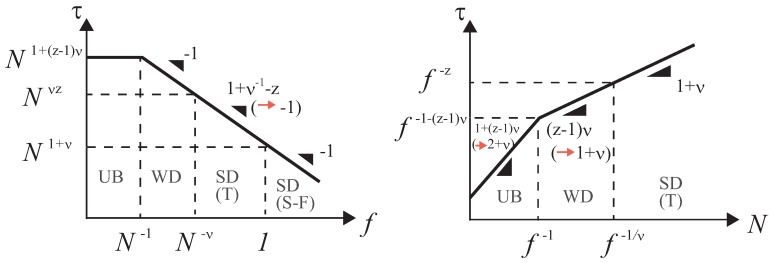
Dependence of the translocation time on *f* (**left**) and *N* (**right**) shown in double logarithmic scale. The locations of various regimes, unbiased (UB), weakly-driven (WD) and strongly-driven (SD) are specified; the SD regime is further divided into the trumpet (T) and the stem–flower (S-F) regimes. Note that in the right graph, depicted is the case with $f<k_{B}T/a$; otherwise, we have only S-F regime with the slope 1+ν. Note also that in these plots, we set $z=2+\nu^{-1}$ (free draining dynamics), in which case the plots become particularly simple. The triangles and their nearby numbers designate slopes (exponents), where the numbers after the arrows specify the values for free draining dynamics. For other choices of the dynamical exponent (i.e., z=3 for nondraining (Zimm) dynamics), the slope in the SD (T) regime in the left graph is changed. The same applies to UB and WD regimes in the right graph. Comparing these plots with Figure 4 in Reference [16], one finds differences in weak force and short chain length regions.

The post-propagation stage follows at $t>\tau_{p}^{\left(f\right)}$ [13]. However, in contrast to the unbiased and the weakly-driven regimes, the propagation stage dominates over the post-propagation stage in the sense that the asymptotic translocation time is predicted to be $\tau ∼\tau_{p}^{\left(f\right)}$ [16,19]. Note that—despite the conspicuous difference in their underlying physics—scaling formulae Equations (35) and (37) are identical in the case of free-draining dynamics $z=2+\nu^{-1}$. If not, they differ in their scaling structures, but one can check a smooth crossover at $f∼N^{-\nu }$. These results on the scaling formulae of the translocation time are summarized in Figure 3.

### 5.2. Discussion

Following the memory effect approach suggested by Panja et al. [10], we have argued $Q_{N}<1$ for the translocation dynamics in unbiased and weakly driven regimes; thus, $\tau ∼\tau_{pp}$ scales differently from $\tau_{p}$. If, on the contrary, one *assumes*$\tau ∼\tau_{p}$ as in Chuang, Kantor, and Kardar [30], one ends up with the different scaling for the anomalous diffusion of the translocation coordinate. At present, there seems to be no definite conclusion on which argument is more appropriate, but let us note the following points on this issue from the literature.

In their Langevin dynamics simulation, de Haan and Slater have demonstrated the increasing impact of the memory effect on the translocation dynamics with the increase in the solution viscosity and the chain length [31,32]. In such a memory-predominant situation, they have shown that (i) the post-propagation stage dominates the translocation time, and (ii) the translocation time scaling is close to $\tau ∼N^{2+\nu }$, which is Equation (35) with $z=2+\nu^{-1}$, in accordance with Panja et al. (see Appendix A.4). However, several reports on the numerically-estimated subdiffusion exponent in the propagation stage do not match the value in Equation (34) [31,32,33]. The reported exponent looks to be closer to the value suggested by Chuang et al. [30].

It should be kept in mind that we have only considered the asymptotic scaling, which would be valid in the long chain limit. For real situations, a significant finite-size effect would come into play. For strongly-driven translocation, the role of pore friction has been recently elucidated [8,17]. Similar effects would be likely for unbiased and weakly-driven translocations as well, and it might be a possible source for the puzzling observation mentioned above.

In Reference [34], the scaling for the anomalous drift

(38) $$\begin{array}{c} \langle n\left(t\right)\rangle ∼{\left(ft\right)}^{\left(1+\nu )/(1+2\nu \right)} \end{array}$$

has been proposed based on the combination of memory effect argument similar to ours and the numerical simulation results. However, this is different from our linear response prediction Equation (18), though the exponent on *t* is the same (note $z=2+\nu^{-1}$ for the free draining dynamics, so the time exponent is (1+ν)/(νz)=(1+ν)/(1+2ν)). Because of this, a scaling formula for *τ* different from Equation (35) was proposed in Reference [34], but this does not seem to provide a clear crossover scenario both to unbiased and strongly-driven regimes.

In conclusion, we have suggested the introduction of the weakly-driven regime for polymer translocation dynamics, which is naturally described by the linear response theory applied to the unbiased regime. A similar discussion on weakly and strongly-driven dynamics for a polymer pulled by mechanical force has been recently done in Reference [35]. We hope the resultant concise crossover scenario will be useful to promote the understanding of polymer translocation dynamics in near-equilibrium situation, which is necessary to unveil a full picture of the phenomenon.

## Appendix A.

### Appendix A.1.

These two critical exponents are useful for the development of a general scenario. The static exponent *ν* (often called the Flory exponent in polymer literature)—being the inverse of the fractal dimension—quantifies the polymer’s spatial extension as a function of its molecular weight. While ν=1/2 for ideal chains, the swelling due to the excluded-volume effect results in a larger value, which depends on the space dimension. On the other hand, the dynamical exponent *z* is associated with the dissipation mechanism at work. In the simplest model (as adopted in many of simulation studies), the motion of monomers caused by the flow of the surrounding medium is neglected. This free-draining dynamics (sometimes called local friction or Rouse dynamics) amounts to set $z=2+\nu^{-1}$. In dilute solution, however, the solvent-mediated hydrodynamic interaction is known to be relevant. The resultant non-draining dynamics can be approximated by setting z=3 (Zimm dynamics), at least, in free space.

### Appendix A.2.

This preprint [20] was prepared for a supplemental to Reference [19]. The latter is an erratum to Reference [13], where we have modified earlier scaling predictions [11,12,13] to be consistent with the appropriate mass conservation relation across the pore, as pointed out in Reference [16].

### Appendix A.3.

This relation is deduced from Equation (30) with the relation m(t)∼n(t), which is expected to be valid asymptotically; see Reference [20].

### Appendix A.4.

Notice, however, that the scaling $\tau ∼N^{2}$ was observed in Reference [31,32] in the opposite limit of low viscosity, where the polymer conformational relaxation is rapid. The same scaling is also observed in the limit of high imposed pore friction [36]; see also earlier works [23,24]. For practical purposes, one may add the solvent viscosity and the pore friction as extra parameters (possibly their combination with the chain length) to determine the appropriate regime.
